# Fostering bioinformatics education through skill development of professors: *Big Genomic Data Skills Training for Professors*

**DOI:** 10.1371/journal.pcbi.1007026

**Published:** 2019-06-13

**Authors:** Yingqian Ada Zhan, Charles Gregory Wray, Sandeep Namburi, Spencer T. Glantz, Reinhard Laubenbacher, Jeffrey H. Chuang

**Affiliations:** 1 The Jackson Laboratory for Genomic Medicine, Farmington, Connecticut, United States of America; 2 Genomic Education, The Jackson Laboratory, Bar Harbor, Maine, United States of America; 3 Center for Quantitative Medicine, UConn Health, Farmington, Connecticut, United States of America; 4 Department of Genetics and Genome Sciences, UConn Health, Farmington, Connecticut, United States of America; University of Toronto, CANADA

## Abstract

Bioinformatics has become an indispensable part of life science over the past 2 decades. However, bioinformatics education is not well integrated at the undergraduate level, especially in liberal arts colleges and regional universities in the United States. One significant obstacle pointed out by the Network for Integrating Bioinformatics into Life Sciences Education is the lack of faculty in the bioinformatics area. Most current life science professors did not acquire bioinformatics analysis skills during their own training. Consequently, a great number of undergraduate and graduate students do not get the chance to learn bioinformatics or computational biology skills within a structured curriculum during their education. To address this gap, we developed a module-based, week-long short course to train small college and regional university professors with essential bioinformatics skills. The bioinformatics modules were built to be adapted by the professor-trainees afterward and used in their own classes. All the course materials can be accessed at https://github.com/TheJacksonLaboratory/JAXBD2K-ShortCourse.

This is a *PLOS Computational Biology* Education paper.

## Introduction

In the past 2 decades, rapid genomic technology development has driven the unprecedented acquisition of biological data and created fundamentally new opportunities for the incorporation of data-driven, computational approaches into life science research. Bioinformatics and computational biology have emerged as new branches of life science that employ mathematical models, statistical analyses, and computational algorithms to integrate, analyze, and interpret data to answer biological questions. In 2016, a Cold Spring Harbor Laboratory survey found that nearly 90% of National Science Foundation (NSF)-funded biological sciences faculty intended to pursue large data set analyses [1]. Accordingly, the job market for bioinformatics scientists is expanding by an average of 2.71% per year [2].

Given the strong demand for bioinformatics scientists, developing educational experiences suitable for a range of skill levels at the intersection of computational science and biology is critical [3, 4]. In the 20 years since Altman first proposed a graduate-level curriculum in bioinformatics [5], integrative and adaptive bioinformatics courses continue to be refined to address the inherently interdisciplinary and fast-evolving nature of the field [3, 4, 6]. In 2014, the Curriculum Task Force of the International Society for Computational Biology (ISCB) Education Committee synthesized and published a set of guidelines for bioinformatics education that have since been widely adopted [7]. Since then the ISCB guidelines have been used to refine bioinformatics programs in the US; however, it is not easy to implement a full-fledged bioinformatics program, especially for universities or colleges, which do not have the array of faculty needed for bioinformatics education.

Beyond the ISCB curriculum guidelines, many additional ideas [4, 8–15] have been proposed to promote undergraduate involvement in bioinformatics; we highlight a few here. The Genomics Education Partnership (GEP, http://gep.wustl.edu) provides research opportunities in genomics for undergraduate students from primarily undergraduate institutions (PUIs) [16]. The NSF-supported Genome Consortium for Active Teaching (GCAT) continues to engage educators working with undergraduates in applied bioinformatics and computational training focused on curricular integration [10, 17]. Some groups have integrated bioinformatics into biology classes by pairing biology students with computer science students to solve bioinformatics challenges [18]. Furthermore, there are community initiatives to actively create and curate bioinformatics modules that can easily be incorporated into current biology courses, some of which are available on CourseSource (http://www.coursesource.org).

Although substantial progress has been made in building bioinformatics programs (degrees, minors, certificates, and workshops) using modern bioinformatics curricula, the higher education system in the US is still not meeting the real-world demand for computationally savvy graduates in biology [1, 19–22]. The Network for Integrating Bioinformatics into Life Sciences Education (NIBLSE) [23] surveyed thousands of life sciences faculty in the US and identified faculty training as a significant remaining challenge [22]. The NIBLSE survey and additional references strongly support the notion that lack of faculty training in the field of bioinformatics, particularly at nondoctoral degree–granting institutions, is the major obstacle toward the integration of bioinformatics into undergraduate biology [3, 19]. To narrow the gap, Rosenwald and colleagues have launched GenomeSolver (http://genomesolver.org) to teach biology faculty to use basic bioinformatics tools [24].

At The Jackson Laboratory (JAX), a nonprofit biological research institute, two active projects increase teachers’ and professors’ ability to integrate 21st century genetics and genomics into life science courses. The JAX Genomic Education group has trained more than 160 high school teachers in a professional development program called Teaching the Genome Generation (TtGG). The program introduces genetics, bioinformatics, and bioethics into high schools [24]. In this manuscript, we present a professional training program that we designed and administered over the last 3 years to deliver an immersive, 1-week skills development course for undergraduate college and regional university faculty from biology, mathematics, and computer science disciplines. This program, *Big Genomic Data Skills Training for Professors*, was supported by the National Institutes of Health (NIH) Big Data to Knowledge (BD2K) project and operated by JAX. Since 2016, we have held four big genomic data courses for faculty in Farmington, Connecticut, at JAX Genomic Medicine (JAX GM) and in Bar Harbor, Maine, at JAX Mammalian Genetics (JAX MG). This course has served faculty from liberal arts colleges and smaller, regional universities across the US. The implicit goal of the training program, herein referred to as JAX BD2K, was to prepare faculty participants to teach big genomic data skills. We have designed and built portable/manageable bioinformatics modules (https://github.com/TheJacksonLaboratory/JAXBD2K-ShortCourse) for the professors to implement as appropriate for their campus cultures and academic programs. We expect that by application of our accessible modules, faculty nationwide with similar needs may better engage students and teach the core concepts of bioinformatics and genomic computational biology.

## Program structure

From 2016 to 2018, JAX hosted a total of 91 course participants (average 23 per session) who were actively involved in undergraduate/graduate education and brought various backgrounds to the course—mostly in biology (*n* = 76), with a few in mathematics (*n* = 3), computer science (*n* = 6), medicine (*n* = 5), and veterinary medicine (*n* = 1). Although participants expressed interest in teaching bioinformatics, no prior knowledge of bioinformatics was required. The NIH BD2K Initiative (R25 EB022365) funded registration and lodging.

The goal of JAX BD2K was to (1) immerse professors from small colleges, regional universities, historically black colleges and universities (HBCUs), and NIH IdEA Network of Biomedical Research Excellence (INBRE) institutions in the practice of big genomic data computational biology and (2) enable participants to launch bioinformatics or computational biology courses at their home institutions. Toward achieving those aims, the 5-day training program included seminars, extensive hands-on data analyses, evening activities, and collaborative curriculum development forums for course planning. The forums provided a unique opportunity for faculty to raise concerns with peers and discuss integration of JAX BD2K modules into their courses. After the conclusion of each BD2K training program, JAX continued to provide support by inviting trainees to join a dedicated Slack communication platform (https://bd2k-jax-org.slack.com) to share ideas and build a peer network around undergraduate bioinformatics training. JAX has also offered ongoing technical support, often required for implementation of hands-on undergraduate bioinformatics training. To encourage collaborations and discussions from the broader community, JAX has deposited all the course materials to a GitHub repository and made them publicly available at https://github.com/TheJacksonLaboratory/JAXBD2K-ShortCourse.

The core JAX BD2K program agenda is listed in Table 1. The course opened with an introduction to essential concepts in big data generation (high-throughput sequencing technologies) and processing/analysis (guided practice on scripting in R/UNIX and essential statistics) (Table 1. Top, blue shade). One day of training was not sufficient for most participants to attain mastery of these topics; however, trainees had opportunities to revisit the material and reinforce their understanding during subsequent lectures and hands-on analysis exercises. Additional training beyond JAX BD2K, especially in the areas of statistics and scripting in R/UNIX, would be useful for novices. The program was not specifically designed with the nine NIBLSE core competencies [4] as a guide; however, all the competencies except C9 (“Interpret the ethical, legal, medical and social implications of biological data”) were covered during each workshop.

**Table 1:** Program structure of *Big Genomic Data Skills Training for Professors*.

	Teaching Focus	Specific Topics
JAX BD2K Program Structure	Basics	High-throughput sequencing technologies
		Statistics
		Scripting in R/UNIX
	Modules	RNA-Seq
		Cancer variant
		Exome
		Microbiome
		ChIP-Seq
	Miscellaneous	Setting up educational cloud and grants
		Curriculum discussion
		Slack discussion community

Top, blue shade: Minimal cornerstones of bioinformatics introduced in the course.

Middle, orange shade: Modules on different data types covered by the 2018 course.

Bottom, green shade: Various topics to assist bioinformatics course development.

Abbreviations: BD2K, Big Data to Knowledge; ChIP, chromatin immunoprecipitation sequencing; JAX, The Jackson Laboratory; RNA-Seq, RNA sequencing.

Hands-on exercises (Table 1. Middle, orange shade) were taught through bioinformatics case studies designed to reflect the range of big genomic data analyses currently popular in the field. Brief module descriptions are presented in Table 2. The conceptual goals of the modules were to allow the professor-trainees to build familiarity with (1) different types of sequencing data, (2) aligning short reads to a reference genome, (3) gene annotation, (4) downstream analyses and visualizations, and (5) how to present and interpret big data. The bioinformatics/process goals of the modules were to have professors learn to run bioinformatics pipelines and gain experience with a variety of analytic platforms and software packages. Each module began with 30–40 minutes of instruction that covered underlying biological concepts and introduced the bioinformatics pipeline that would be used. Then, trainees worked through approximately 2 hours of guided hands-on data analysis practice. Importantly, research data sets addressed modern biological questions yet were scaled such that the required computations could proceed with limited technical resources and could complete within a typical college class schedule. Modules were designed to consist of discrete analysis steps that could be adopted directly by trainees or customized by the selective omission or addition of one or more analyses. For example, the RNA sequencing (RNA-Seq) module allows for optional gene set enrichment analysis or may be appended with exercises for building gene networks for system biology analysis depending on student needs and learning goals. To enable participants to revisit modules at a later date, each hands-on session was accompanied by a manual containing background information, a detailed listing of analysis steps, and extensive explanation.

**Table 2:** Description of data analysis modules in *Big Genomic Data Skills Training for Professors*.

Modules	Skills	Biology Question	Platform	Data Source
**RNA-Seq**	Differential gene expression analysis and gene set enrichment	What are genes affected by Pax6 knockout in male mice?	Galaxy	Mitchell and colleagues 2017
**Cancer Variant**	Data manipulation—grouping and sorting	What is the common driver mutation in three melanoma tumors?	Excel/R	Berger and colleagues 2012
**Exome**	Variant calling and filtering	Identify the exonic variant and gene responsible for the phenotype of “Leg dragger”* in mice.	Galaxy	Fairfield and colleagues 2011
**Microbiome**	16S analysis and bacterial taxon cataloging	What is the role of the microbiome in the development of type 1 diabetes in infants?	R/UNIX	https://pubs.broadinstitute.org/diabimmune
**ChIP-Seq**	Peak calling and motif analysis	Identify CTCF binding motif.	UNIX (Cloud)	ENCFF000ARV, ENCFF000ARP, ENCFF000ARK

*“Leg dragger” is a spontaneous mutation leading to a phenotype where the mouse drags its rear legs and pulls it along with its front legs to move.

Abbreviations: ChIP-Seq, chromatin immunoprecipitation sequencing; RNA-Seq, RNA sequencing.

Alongside basic knowledge and skills training, the agenda provided time for curriculum discussion forums. These were convened three times—at the beginning, halfway, and on the closing day of the training week (Table 1. Bottom, green shade). Trainees benefited from the discussion with their peers for conceptualizing curricular designs and anticipating obstacles faced in their departments or by their students. To serve faculty with insufficient information technology (IT) support at their home institutions, the JAX BD2K program also provided training on the setup of educational cloud computing resources/service and guidance on grant opportunities available for cloud computing. Beyond the program components covered in Table 1, we also hosted a number of seminars on diverse research programs (S1 File) that employ bioinformatics analyses to highlight the applications of bioinformatics in modern research. Speakers were from JAX and UConn Health (the medical school of the University of Connecticut).

## Preliminary evaluation of JAX BD2K program

Prior to the beginning of the course, all participants were surveyed in a 10-item questionnaire about their bioinformatics knowledge, expertise, interests, and computation resources at their home institutions. After completing the week-long training, participants were given a postevent survey/questionnaire with 22 questions asking their opinions on the lectures and modules in the course and their suggestions for improvement. These were a mix of questions on a numerical scale and questions with open-ended responses. Questions from the questionnaires for the 2018 workshop can be found in the S2 and S3 Files. Although the course contents varied every year, the survey questions mostly remained the same except for some specific ones on individual modules. In the following sections, we report aggregated results from the past 3 years for some generic questions. For the questions only concerning one workshop, we focused the analysis for that workshop only.

## Preevent survey

From 2016 to 2018, we catered to 91 professors from 78 universities in the US and one in Canada. A list of participating institutions can be found in the S4 File. (https://tinyurl.com/JAX-BD2Kprof). Participants were selected by the organizing team, which includes two computational biology faculty members (Chuang, Laubenbacher) and one professional education specialist at JAX (Wray). Professor-applicants were asked to list the undergraduate courses they teach, describe their interest in integrating genomic big data topics into their courses, and provide information about their research efforts. Preference was given to applicants that primarily teach undergraduates at small colleges or regional universities (Carnegie-defined master's colleges and universities and baccalaureate colleges).

**Fig 1:**
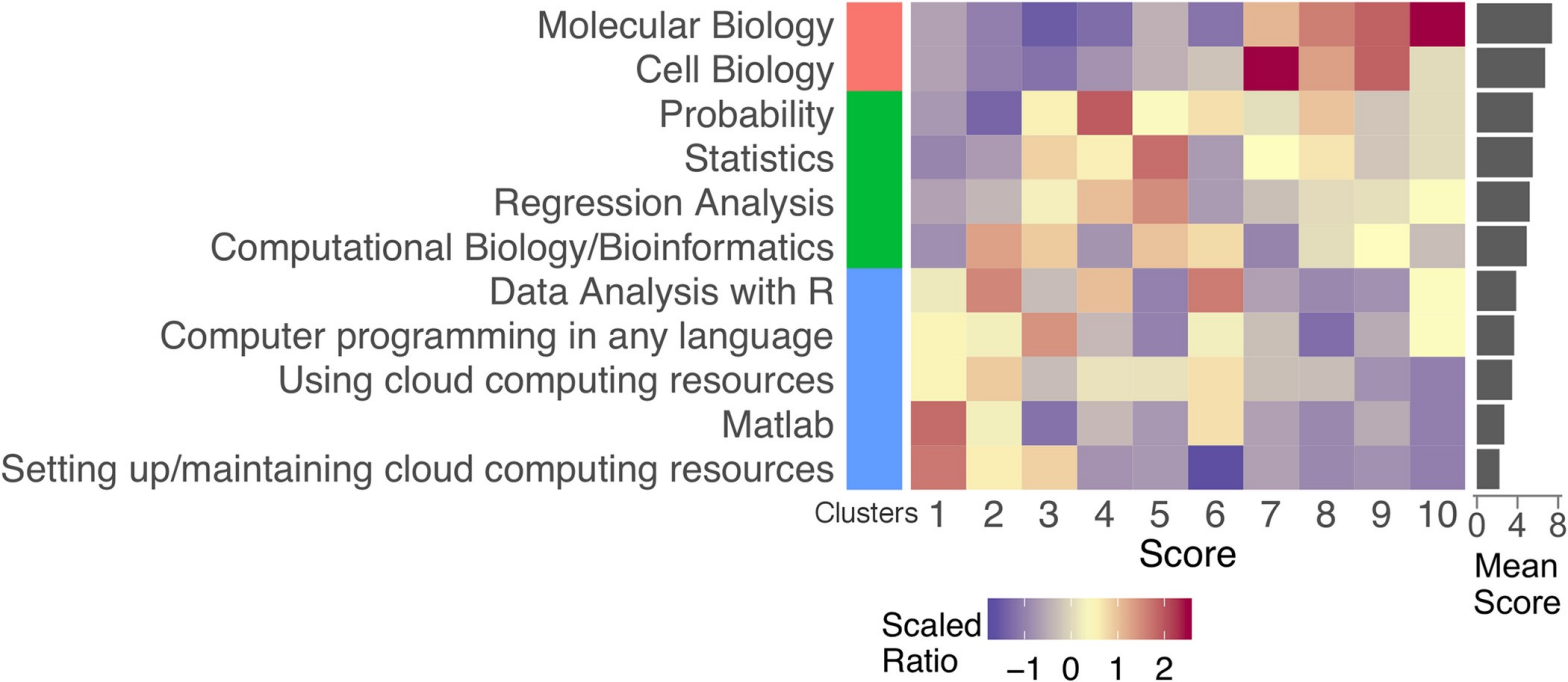
Participant profile of *Big Genomic Data Skills Training for Professors*. Background knowledge survey on 11 subjects. The expertise on each subject was evaluated from 1 (no knowledge) to 10 (expert). The mean score of each subject is shown at the right. The respondent ratio for each subject at every score is scaled to the center of each row and shown in color (middle panel). K-means clustering was conducted on the respondent ratio and presented to the left of the score matrix.

Prior to the training event, we conducted a voluntary, anonymous 10-question survey to assess professor-participants’ knowledge background, course expectations, and computation capabilities at their home institutions. The professors were from biology (83.4%), medical sciences (5.5%), computer science (6.6%), mathematics, or statistics backgrounds (3.3%). Regarding their knowledge background (Q2 in S2 File: “From 1 (no knowledge) to 10 (expert), rate your expertise on each of the following”), we recorded 45 out of 91 responses in 3 years, and the aggregate results are shown in Fig 1. “Molecular Biology” and “Cell Biology” were the two most highly scored knowledge areas, suggesting that the responding pool contains essentially biologists, whereas “Setting up/maintaining cloud computing resources” claimed the lowest score (Fig 1. Mean Score panel). To clarify the knowledge structure, we performed k-means clustering on the subjects in Q2 of S2 File based on the ratio of respondent counts at each score (Fig 1. Middle panel: ratios are scaled by the center of each row). Interestingly, we observed three clusters: cluster 1 contains two biology subjects with the highest scores; cluster 2 includes four theoretical subjects on statistics and computation with spread out scores; and cluster 3 is a group of practical computation skills in which the respondents claimed “not very proficient” with (Fig 1. Clusters panel). Overall, the majority of the participating professors were experienced biologists with some level of genetics and genomics knowledge but a lack of practical skills in computation, consistent with our expectation that this is the most common group being tasked with administering bioinformatics courses around the US. One encouraging sign is that 26 out of 44 (~59%) respondents admitted computing cluster accessibility to the students, and 38 out of 45 (~84%) indicated that IT support existed at their home institutions, suggesting computational infrastructure may not be a key hurdle to integrating big genomic data skills training at PUIs. Although such access to IT support is encouraging, setting up the cloud computing infrastructure necessary to teach some program content may be beyond the expertise of IT support staff at many PUIs.

## Postevent survey

To evaluate the JAX BD2K training program, we designed a 22-question postevent survey to gather participant feedback regarding satisfaction, module/seminar evaluation, self-evaluation on learned knowledge, and comments/suggestions for improvement. An example survey from the 2018 workshop is provided in S3 File.

**Fig 2:**
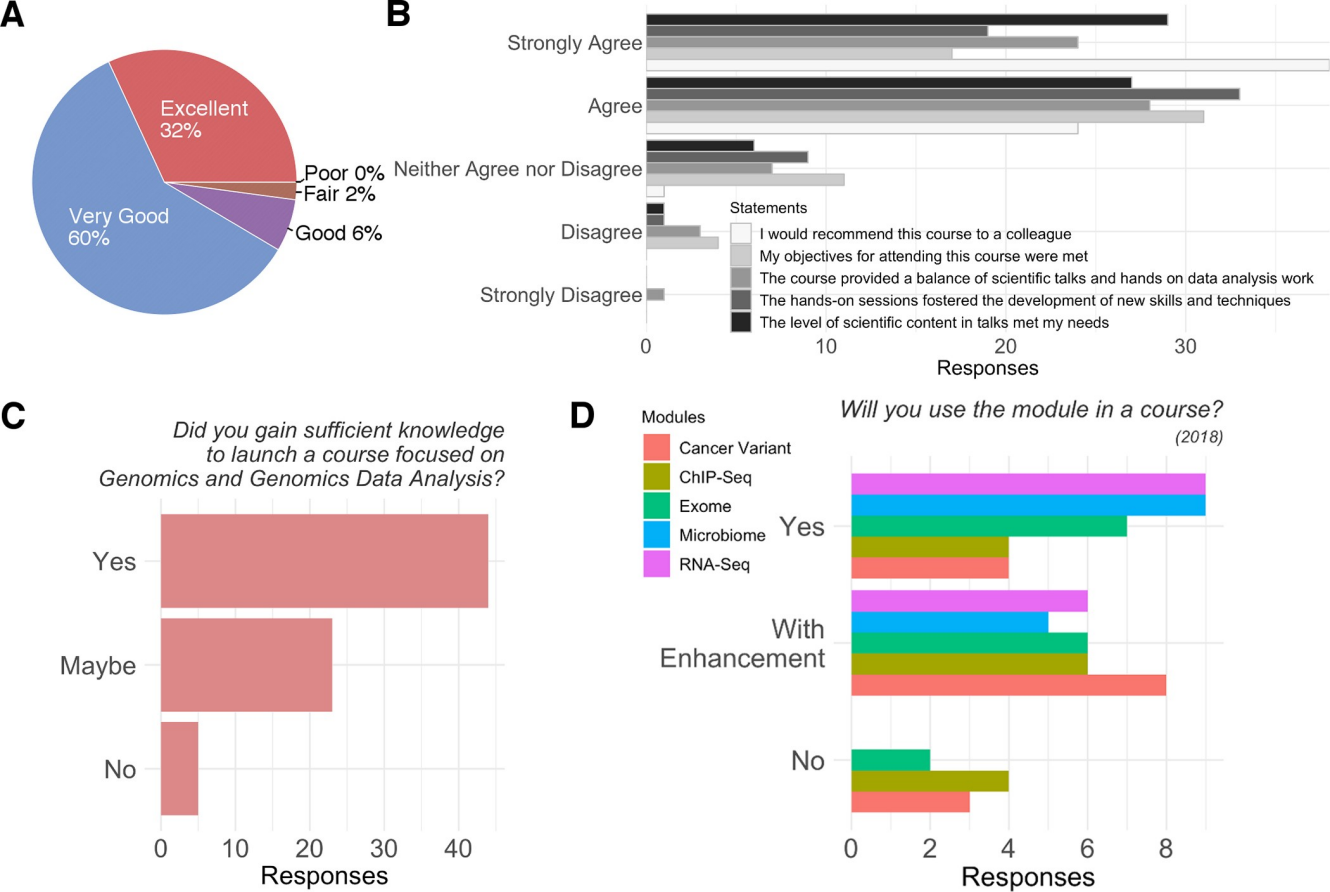
Postevent survey on the satisfaction and evaluation of *Big Genomic Data Skills Training for Professors*. (A) Participants’ overall satisfaction on the training program. (B) Distribution of degree of agreement on specific statements regarding the program. (C) Distribution of answers to their confidence to launch a course focused on genomics and genomics data analysis. (D) Distribution of participants’ willingness to implement our modules to their course. ChIP-Seq, chromatin immunoprecipitation sequencing; RNA-Seq, RNA sequencing.

Overall, participants gave very positive feedback. Among 47 out of 91 responses, 32% rated their training as “Excellent” and 60% as “Very Good,” and no participants regarded their experience as “Poor” (Fig 2A). Participants were invited to give opinions on a list of statements related to their overall feelings on the program as well as satisfaction levels on individual aspects. In Fig 2B, we present their judgements with some general comments on the program. For this voluntary inquiry, 63 out of 91 participants from the past 3 years responded. Thirty-eight responders strongly agreed that they “would recommend this course to a colleague,” 33 responders agreed that “the hands-on sessions fostered the development of new skills and techniques,” and 31 agreed that their “objectives for attending this course were met.” The other two statements, i.e., “The course provided a balance of scientific talks and hands-on data analysis work” and “The level of scientific content in talks met my needs,” were also highly rated in either the “Strongly Agree” or “Agree” categories. Survey questions that queried participants regarding specific course design elements (S3 File Q4 and Q6), such as instruction and assistance, were scored highly in the “Agree” and “Strongly Agree” categories as well.

The ultimate goal of JAX BD2K is to promote bioinformatics and computational learning among undergraduates. Therefore, we were interested in ascertaining participants’ willingness and intention to implement JAX BD2K modules in their classes. Because all participants had minimal formal training in bioinformatics, we were uncertain how confident they would be to launch a course involving bioinformatics data analysis. Of the 72 (out of 91) participants that answered the question “Did you gain sufficient knowledge to launch a course focused on Genomics and Genomics Data Analysis?” over 61% (44 out of 72) of trainees responded affirmatively. Developing and launching an undergraduate course does not happen rapidly, but a pool of confident educators is a promising start.

Hands-on data analysis modules are a critical element of the JAX training program. For the first incarnation of the JAX BD2K program in 2016, the hands-on sessions were not module based. The participants of the first version of the JAX BD2K course urged us to develop hands-on modules including short lectures, hands-on sections, and step-by-step manuals. In the following year, we created five modules, but three of them involved processing full genomes, which was not practical in most undergraduate classrooms, because of limited computational resources. In the most current version of JAX BD2K, we scaled down all the data sets, included more detailed, stepwise data analysis instruction, and centered the analyses around specific biological questions. Every module is designed to complete within 2 hours on a Galaxy public server, one compute node with eight processors, or a personal computer. All the instructions and steps of analyses can be delivered to students one at a time, which would give instructors more flexibility on how the course should be run. We asked our participants about their willingness to implement these newly optimized modules. Of 15 responders (out of 23 participants in 2018), 9 stated they would directly implement in their current form the RNA-Seq and Microbiome modules, the two most popular hands-on exercises conducted (Fig 2D). Roughly one-third of the responding pool had interest in implementing any of the modules after additional optimization (except for the Cancer Variant module). Overall, satisfaction with the 2018 module versions was high.

Receiving individual comments and suggestions via postevent surveys has been indispensable for our iterative refinement of the JAX BD2K course. We designed five open-ended questions to gather participants’ opinions on how we can improve, what to remove, add, or flesh out, and how to mix hands-on work with didactic sessions more effectively. During the past 3 years, comments/suggestions have shifted from “more hands-on” (2016) and “written instruction” (2016) to “few of the modules worked” (2017) to now “more defined modules to undergraduates pedagogically” (2018). At the same time, our program evolved from less-structured hands-on work to adaptable modules. Based on the feedback we received, we are continuing to improve our modules and develop new ones. All updated material will be posted on Slack and GitHub and subject to testing and implementation.

## Pilot results on implementation of JAX BD2K modules

From past alumni, we learned that professors have successfully integrated some of the training modules listed in Table 2 into their undergraduate courses. The RNA-Seq module is popular and has been used at regional universities in Arkansas, Tennessee, and Oklahoma. Two small colleges in New England launched new bioinformatics courses after faculty trained in the JAX BD2K program. At Colby College, the new course included a faculty-built RNA-Seq module that targets 15 genes on mouse chromosome 5 wherein undergraduates who are novices in genomic data analysis can learn and complete an RNA-Seq workflow in two afternoon lab sessions. The program also helped a participant institution earn a new NSF grant that will offer workshops and hackathons at three PUIs/HBCUs in Tennessee, Georgia, and Alabama.

## Computational platform

For computing and storage, we deployed a private instance of Galaxy CloudMan [24] on Amazon Web Services (AWS). Galaxy CloudMan [24] provides an easy way to deploy Galaxy with the preinstalled tools on the cloud resources. AWS provides on-demand computing and storage resources with a pay-for-use usage model.

We leveraged AWS's elastic cloud computing capabilities to provision computing and storage resources to minimize costs. Galaxy CloudMan [24] provides a feature to scale the number of compute instances (AWS EC2) based on the usage and capacity. During the workshop, the compute resources were manually increased without relying entirely on the Galaxy CloudMan's autoscaling feature, as the instances take some time to initialize and accept jobs from the queue. For the workloads that needed command-line access, we assigned an instance to each of the participants. All the instances were preconfigured with tools and data.

## Discussion

Professor-participants were highly satisfied with the training program according to the postcourse survey feedback. Yet there are several caveats we learned from the program that will inform future iterations of the JAX BD2K program. Professors were a mix of beginners to sophisticated bioinformatics users, and it has been challenging to cater to such a broad spectrum of adult learners. For beginners, entry-level computational skills are vital and desired; for instance, one comment in a postevent survey read, “R/software/package installation was not sufficiently covered.” Other more experienced participants were looking for learning advanced algorithms, like “Hidden Markov Models” and “dimensional reduction,” and updating with cutting-edge technologies, like “single cell” and “machine learning.” A number of postevent survey comments suggested “pre-attendance homework for coding” or “pre-workshop assignments associated with … R and command line.” These precourse learning modules might help close the gap across the participant knowledge spectrum; however, essentially all the participants teach and lecture as their dominant institutional role, and precourse online learning might not be a seen as achievable to faculty as they complete their teaching semesters. Perhaps a better solution is to divide the participant group based on their computational needs and create modified workshop programs for each group. From the feedback in the postevent survey, we learned that the need for knowledge updates is also prominent. This point was also highlighted in a number of surveys summarized by Attwood and colleagues [3]. Because bioinformatics is a fast-moving field, keeping up with new technologies is a persistent challenge for both professionals and educators. Another suggestion made by a program attendee reads, “The participants always looked at a tutorial in the context of being able to deliver that module to undergraduates pedagogically, rather than just understand and follow the workflow themselves. It might be better to have each module section co-taught by an experienced PUI genomics faculty member (pedagogical expert) paired with a JAX (content) expert in order to get the best of both worlds so to speak.” Because some of our speakers are research scientists who may have limited experience teaching undergraduates, it might be helpful to at least have a pedagogical advisor to help develop the modules and coordinate with speakers on their lecture contents.

Although Galaxy CloudMan [24] was easy to set up and configure, the administration is a time-consuming process. Depending on existing expertise or IT security policies, it may be necessary to have institutional IT or a system administrator’s support to set up the AWS account and install the necessary software on Galaxy and the AWS instances prior to the workshop. During training workshops at JAX, a dedicated person was needed to launch, manage, and monitor the AWS instances. In some scenarios, when a cloud computing platform is not possible, we would recommend use of IT support to ensure compatibility of the infrastructure and software. Public Galaxy is a good option if running time is not a big concern. It is free, has a list of preinstalled tools, and provides timely software support. In undergraduate courses led by JAX staff, students have success submitting computationally intensive portions of a workflow and subsequently learning other materials while a job runs on public Galaxy. We would also encourage readers to explore other cloud resources and funding mechanisms as discussed previously by Langmead and colleagues [24].

## Conclusion and outlook

In the past 3 years, our JAX BD2K short course for training faculty in teaching computational biology/bioinformatics has been proven to be a desirable program. Ninety-one professors from liberal arts colleges and smaller, regional universities across the US have been trained. We have developed adaptable bioinformatics modules to be implemented appropriately into an undergraduate curriculum. We expect that through our modules students may learn basic concepts about what bioinformatics is and how bioinformatics works.

In the future, we need to continue updating our training modules and lectures and to create new modules for the rapidly emerging technologies in the genomics field. Although our overall satisfaction has been high, we will continue to enhance our pedagogy to make it easier for professors to more directly incorporate the exercises they practiced at the JAX BD2K program into their classes. Because of the fast-developing progress of this field, new pipelines and new topics will be important to add into the existing curricula.

Because our most recent session finished only several months ago, it is early to assess the long-term impact of the training program. We plan to follow up with our alumni in 2 years for applications of their training to the teaching they do at their home institutions. At the same time, we will maintain communication with them through our Slack and GitHub platform to provide continuous support. We hope that our sharing of our experiences with the community will inform colleagues, encourage collaborations and discussions, and most beneficially, inspire new approaches to enhance bioinformatics education for our field.

## Supporting information

S1 File A sample program schedule. This is the schedule used at *Big Genomic Data Skills Training for Professors*, *2018*.
(PDF)

S2 File Preevent survey. This is the preevent survey of *Big Genomic Data Skills Training for Professors*, *2018*, including questions and answers.
(PDF)

S3 File Postevent survey. This is the postevent survey of *Big Genomic Data Skills Training for Professors*, *2018*, including questions and answers.
(PDF)

S4 File A list of participating institutions. This file contains all participating institutions to *Big Genomic Data Skills Training for Professors* from 2016 to 2018.
(PDF)
