# Supplementary material for: Fostering bioinformatics education through skill development of professors: Big Genomic Data Skills Training for Professors
Source: PLoS Comput Biol. 2019 Jun 13;15(6):e1007026. doi: 10.1371/journal.pcbi.1007026 (PMC6563947; doi:10.1371/journal.pcbi.1007026)
Supplement: S1 File — This is the schedule used at Big Genomic Data Skills Training for Professors, 2018. (PDF) [file pcbi.1007026.s001.pdf]

Big Genomic Data Skills Training for Professors

The Jackson Laboratory for Genomic Medicine (JAX-GM)
Farmington, Connecticut

May 21-25, 2018

SAMPLE SCHEDULE

Monday, May 21

**8:00am**	**Registration**
**8:30am**	**Welcome and Introduction**
**9:00am**	**Introduction to Data Science with Biological Emphasis**
**10:00am**	**Introduction to High Throughput Sequencing Technologies**
**11:00am**	**Hands on Work: Setting up Computing Infrastructure and Introductions to Galaxy, Genome Space, IGV, Gene Pattern, R, and Slack. Setting up of basic tools.**
**12:00pm**	**Lunch**
**1:00pm**	**Introduction to Linux**
**2:30pm**	**Essential Statistical Analysis**
**3:30pm**	**Break**
**4:00pm**	**Curricular Discussion – Presentation and Discussion of UG courses in Genomics, presentation of previous and existing syllabi**
**5:00pm**	**RNA-sequencing Seminar**
**6:00pm**	**Light Dinner**
**7:00pm**	**Evening Lecture**

Tuesday, May 22

**8:30am**	**RNA-sequencing Introduction**
**9:00am**	**Module #1: RNA-sequencing as Measurement Tool; Hands-on Work**
**10:00am**	**Break**
**10:30am**	**Module #1: Hands-on Work continued**
**12:00pm**	**Lunch**
**1:00pm**	**Module Debrief: Lessons learned, Q & A.**
**1:30pm**	**Gene Set Enrichment and Pathway Analysis**
**2:30pm**	**Hands-on Work; Advanced and Derived RNAseq Analysis**
**4:00pm**	**Break**
**4:30pm**	**Module #2: Variant Discovery in Genomic Sequence; Hands-on Work; Mutation and Functional Analysis in R**

<u>Wednesday, May 23</u>

**8:30am**	**Debrief on Module #2 Variant Calling: Lessons learned, Q & A.**
**9:00am**	**Exome Sequencing for Variant Discovery**
**10:00am**	**Module #3: Exome Sequencing Variant Discovery in Galaxy**
**12:00am**	**Lunch**
**1:00pm**	**Introduction to Microbiome**
**2:00pm**	**Hands-on Work: Microbiome**
**3:30pm**	**Break**
**4:00pm**	**Curricular Discussion #2**
**4:30pm**	**Cloud Resources and Galaxy**

<u>Thursday, May 24</u>

**8:30am**	**Data in Context**
**9:30am**	**Network Modeling**
**10:30am**	**Network Modeling short exercises**
**12:00am**	**Lunch**
**1:00pm**	**Data Standards and Best Practices Data Problems: When bad things happen in genomic analysis**
**2:00pm**	**Introduction to ChIP-seq**
**4:00pm**	**Break**
**4:30pm**	**Module Debriefing on Exome, Microbiome, Network, and ChIP-seq; Open Discussion on Participant Needs and Data**
**5:30pm**	**Reception Dinner**

<u>Friday, May 25</u>

**8:30am**	**Integrated Modeling of Genetic and Genomic Data**
**9:30am**	**Running UG Courses in Genomics**
**11:00am**	**Cloud Environments and Web Service Grants**
**12:00pm**	**Lunch**
**1:00pm**	**Collaborative Discussion, Next Steps, and How to Support UG Implementations**
