# Supplementary material for: Fostering bioinformatics education through skill development of professors: Big Genomic Data Skills Training for Professors
Source: PLoS Comput Biol. 2019 Jun 13;15(6):e1007026. doi: 10.1371/journal.pcbi.1007026 (PMC6563947; doi:10.1371/journal.pcbi.1007026)

Q1 - From 1 (least) to 10 (highest), rate your knowledge of/familiarity with each of the following:

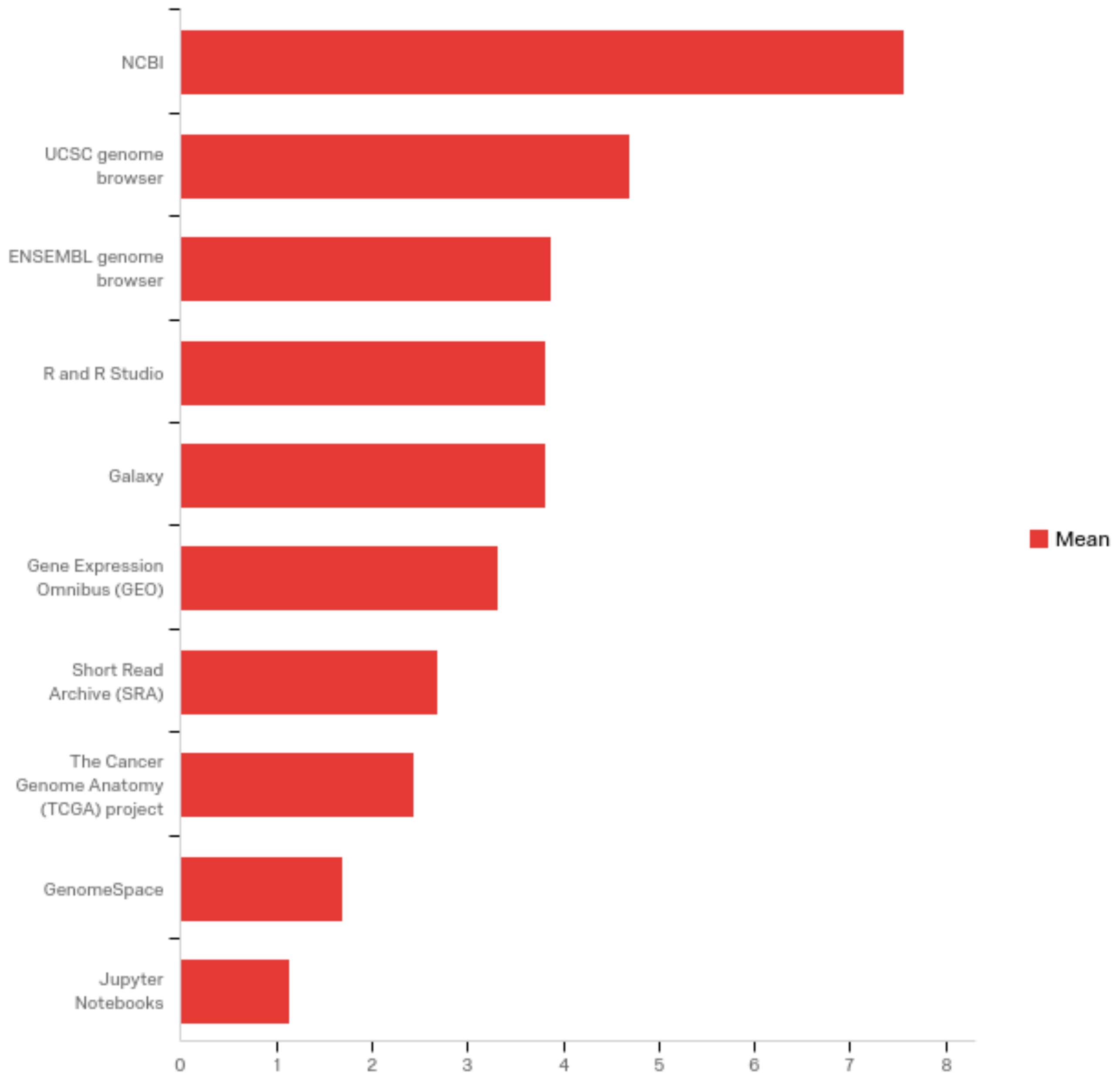

Q2 - From 1 (no knowledge) to 10 (expert), rate your expertise on each of the following:

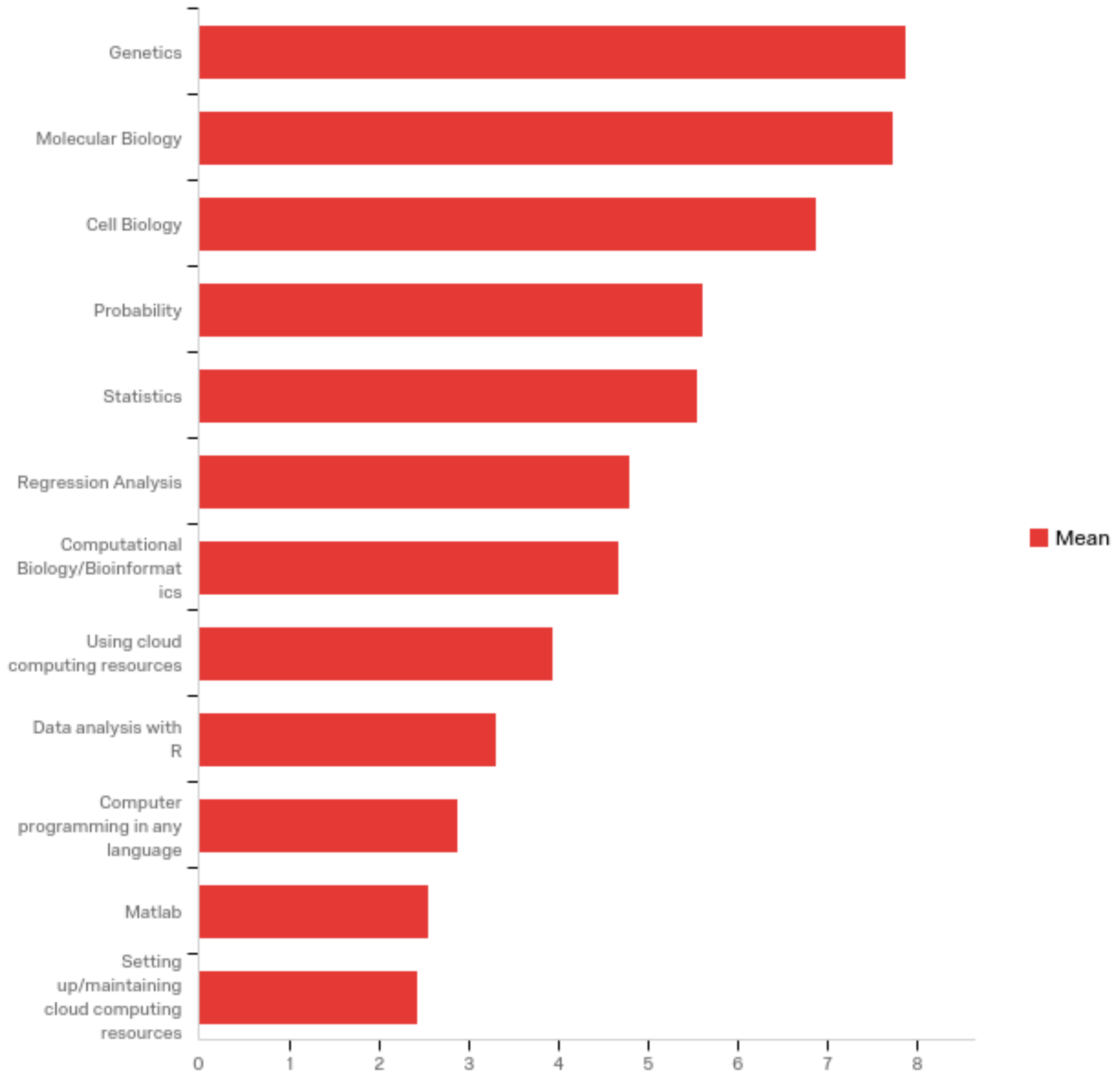

Q3 - Which of the following terms could you define without the use of Google or another search engine (select all that apply):

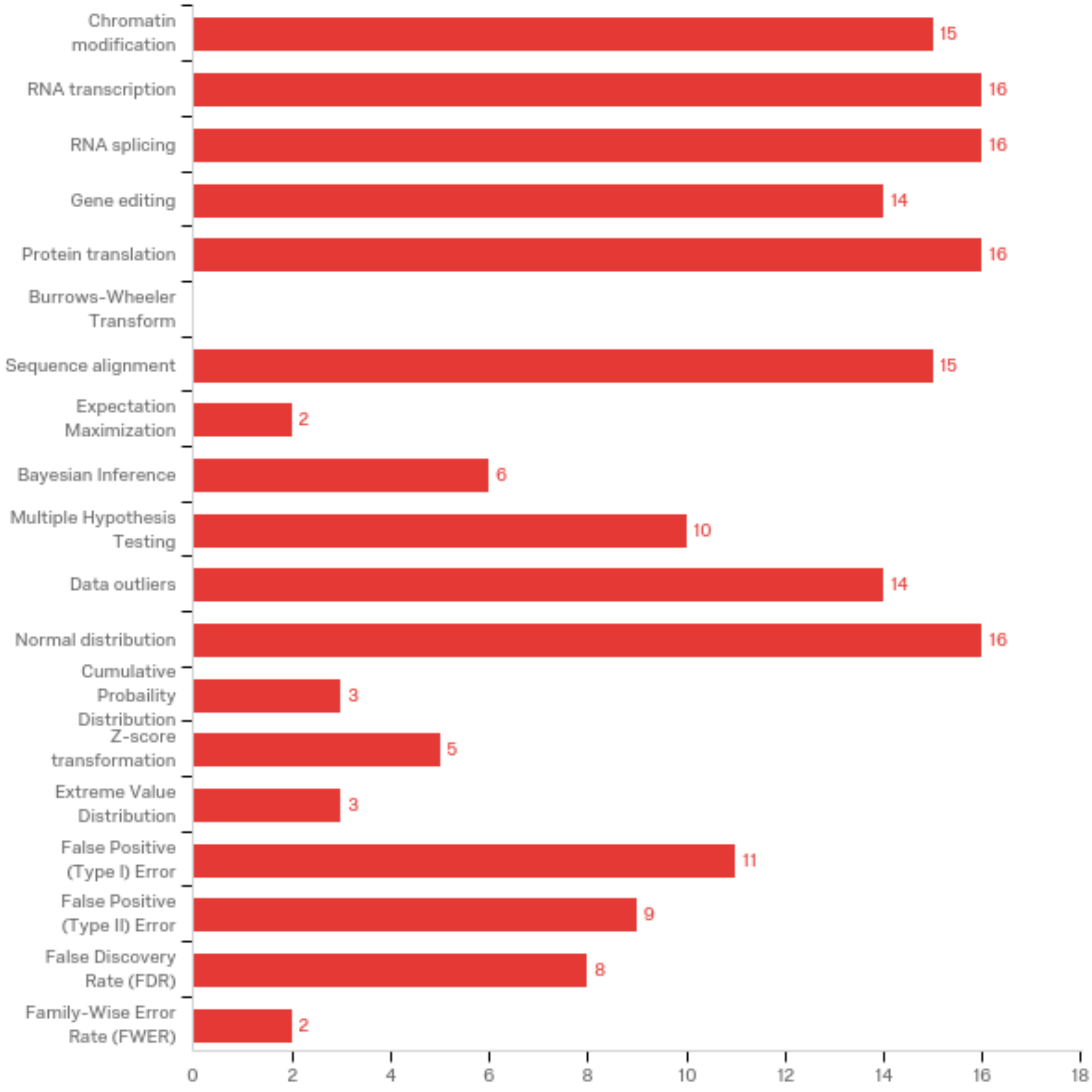

Q4 - From 1 (not interested) to 5 (very interested), rate your interest in learning to work with the following data types. Please select N/A if you are not familiar with the data type.

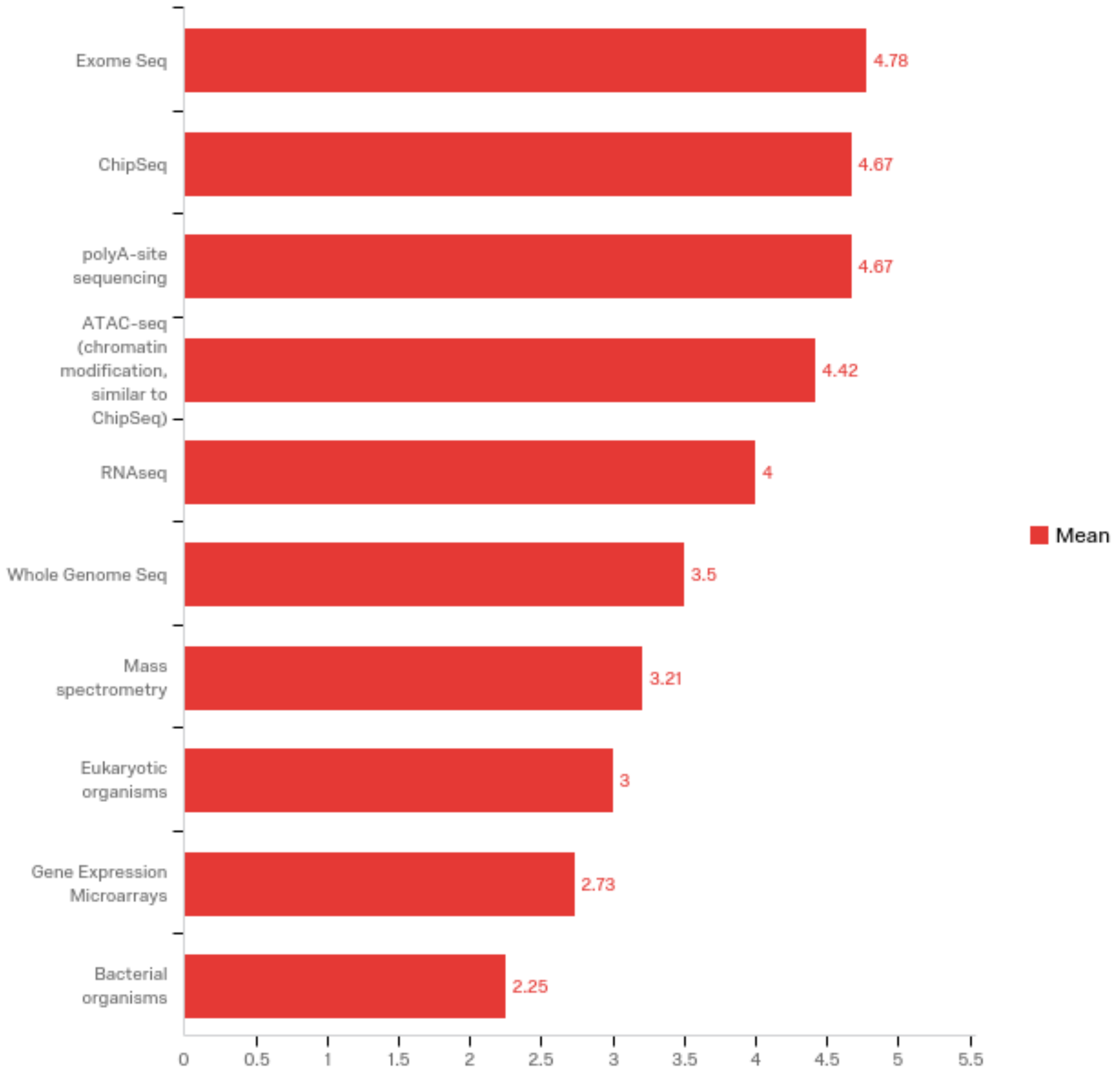

Q5 - Which are you more interested in data generated from:

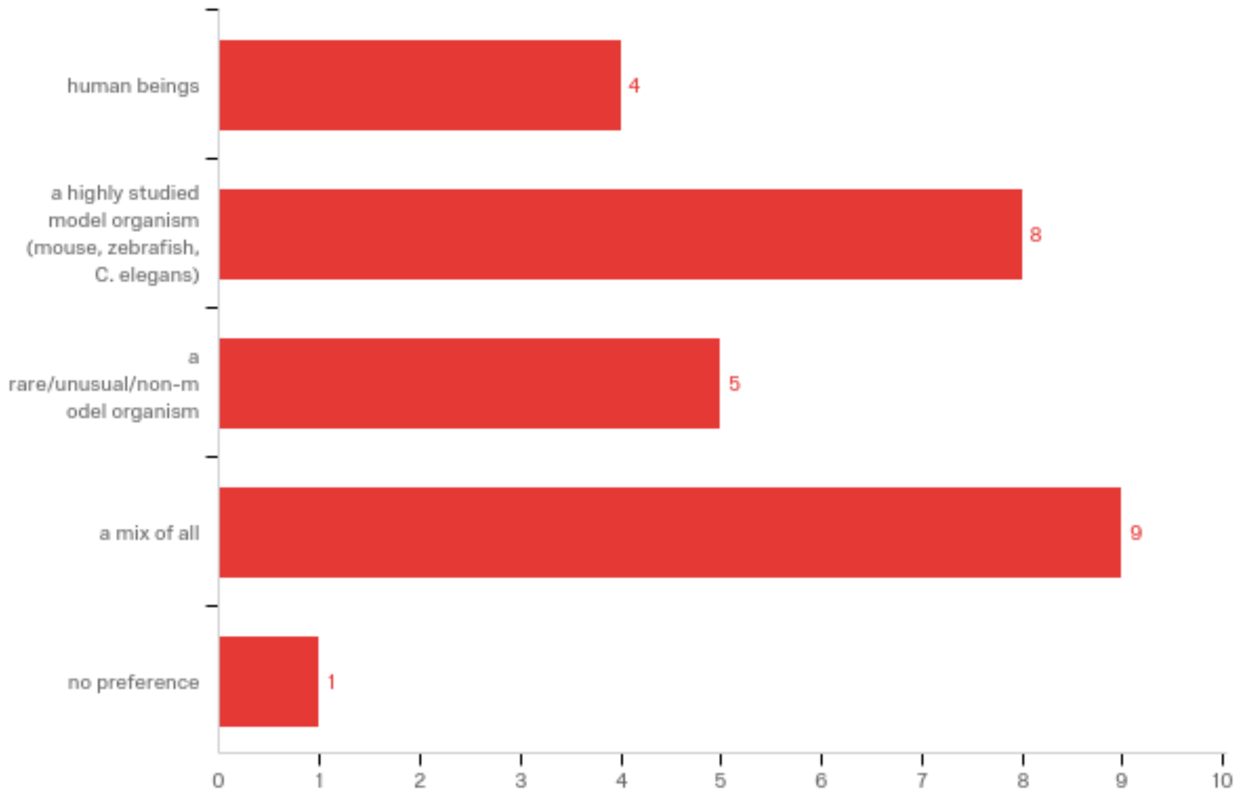

Q6 - Do you have genomic data you might bring to the course?

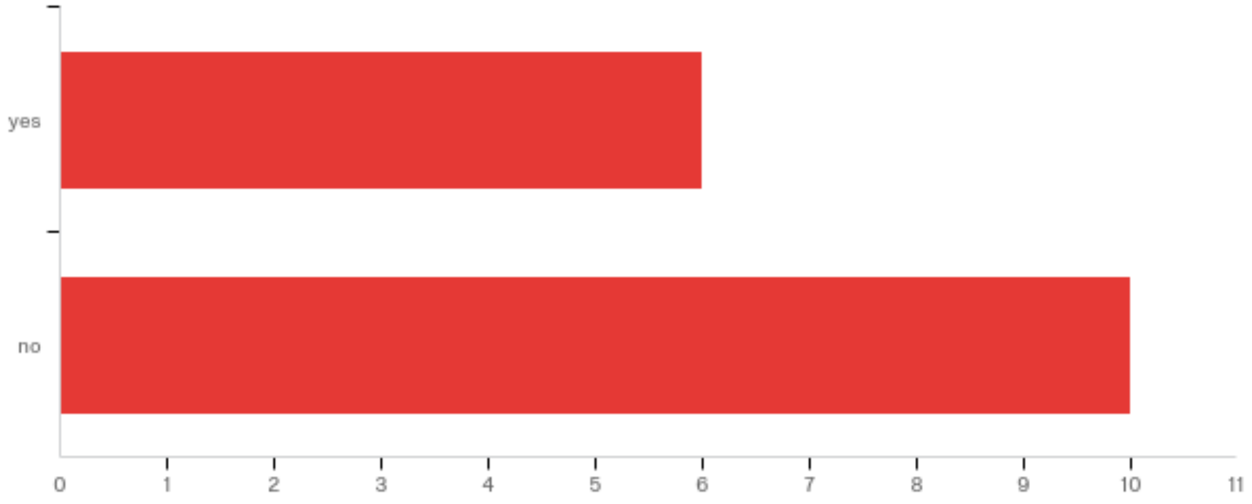

Q7 - Please describe data you may bring to the course:

I have soft-shell clam genomic data in Galaxy
RNA Seq data from mouse eye treated with NaOH +/- transcription inhibitor
I have several bacterial (Burkholderia) genomes I could bring.
I have proteomic data but not genomic data currently available
Drosophila melanogaster brain RNAseq data
Shotgun Metagenomics and 16S marker gene metagenomic data sets
I have 2 data sets comparing treated and untreated cancer lines and looking at transcript expression using Affymetrix GeneChip Human Transcriptome Arrays 2.0

Q8 - Do you have access to a computing cluster that students will be able to login to and perform calculations?

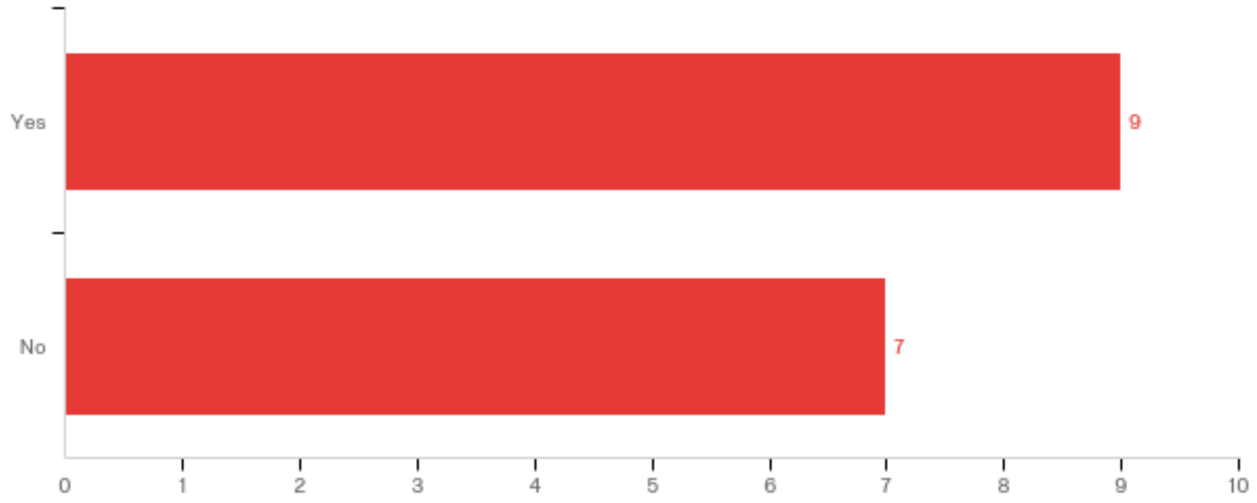

Q9 - Does your college or university have dedicated IT support that can assist you with activities such as software installation?

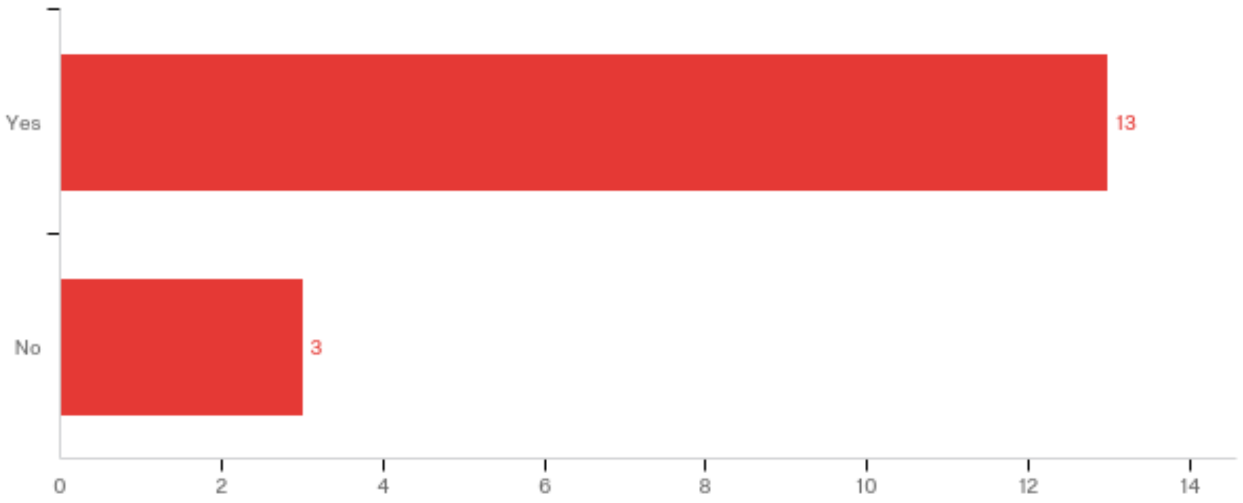

Q10 - Do you have access to teaching assistants to help you mentor students through computational lab activities?

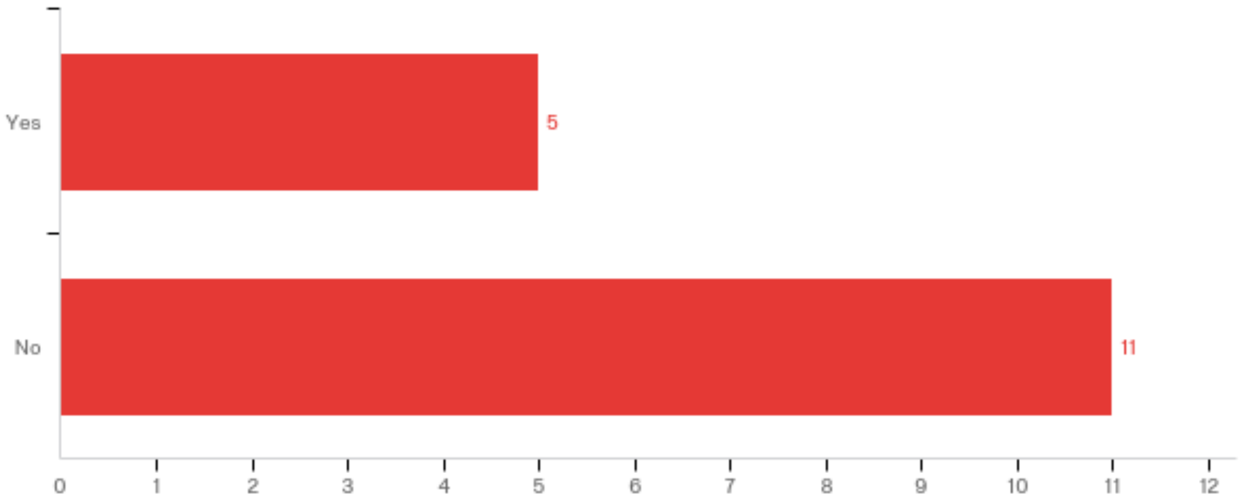

Supplement: S2 File — This is the preevent survey of Big Genomic Data Skills Training for Professors, 2018, including questions and answers. (PDF) [file pcbi.1007026.s002.pdf]
