# Supplementary material for: Fostering bioinformatics education through skill development of professors: Big Genomic Data Skills Training for Professors
Source: PLoS Comput Biol. 2019 Jun 13;15(6):e1007026. doi: 10.1371/journal.pcbi.1007026 (PMC6563947; doi:10.1371/journal.pcbi.1007026)
Supplement: S3 File — This is the postevent survey of Big Genomic Data Skills Training for Professors, 2018, including questions and answers. (PDF) [file pcbi.1007026.s003.pdf]

2018 JAX BD2K Post-event Survey *

Q1 - Overall, how would you rate this course?

#	Answer	Count
1	Poor	0
2	Fair	0
3	Good	0
4	Very Good	5
5	Excellent	5
	Total	10

* In open-ended questions, names of individual staff wherever mentioned were replaced by XXX to protect their identities.

Q2 - To what extent would you agree with the following statements:

#	Question	Strongly Disagree	Disagree	Neither Agree nor Disagree	Agree	Strongly Agree	Total
1	I would recommend this course to a colleague.	0	0	0	6	9	15
2	My objectives for attending this course were met.	0	0	3	5	7	15
3	The course provided an balance of scientific talks and hands on data analysis work.	0	0	1	6	8	15
4	The hands on sessions fostered the development of new skills and techniques.	0	0	0	7	8	15
5	The level of scientific content in talks met my needs.	0	0	1	8	6	15

Q3 - Do you have any comments regarding the usefulness of specific seminar session content?

The systems biology content was over my head... I would be interested if others thought it was useful. I was hoping for information on making co-occurrence style networks with gene expression for data visualization.
I thought it was all very relevant, appropriate for all degrees of learner, and was very useful. Access to the materials are invaluable.
XXX's model was incredibly well designed and presented.
i felt that there could have been less systems/network/ontology content (though I'm very interested in general in epistemology) and more technical and hands-on content. In particular i'd like to have used both R and Galaxy more, and gotten some experience in Jupyter as well.
Very interesting!
I thought Monday-Wednesday were fantastic almost completely across the board. Thursday was a little too dense with lecture and not actually hands-on for me. In particular, I thought XXX really missed the mark with both talks. The content in that case was not deep enough and not helpful above what we'd already heard from XXX the day before. Conversely, while XXX's talk on Friday was very deep, there was enough in to relate to what we'd already talked about that it was incredibly useful.

Q4 - To what extent would you agree with the following statements:

#	Question	Strongly Disagree	Disagree	Neither Agree nor Disagree	Agree	Strongly Agree	Total
1	Sufficient time was provided during talks for questions and dialogue.	0	1	1	7	6	15
2	This workshop promotes professional interchange among participants.	0	0	0	2	13	15
4	Instructors were accessible and responsive to questions outside of sessions.	0	0	0	3	12	15
5	Overall, I was satisfied with the quality of instruction.	0	0	0	9	6	15

Q5 - Do you have any comments regarding the quality of instruction from any course faculty?

I had a hard time following the presentations by XXX and XXX. I have no doubt that they are great scientists, but they need to improve their didactic and communication skills. In contrast, the presentation by XXX was the best one in my opinion.
In some of the tutorials, the Instructor made assumptions or moved to fast for the participants to follow along on their computers.
A number of the speakers were professional researchers, which was great from a knowledge context, but a subset of the speakers lacked well developed presentation skills and made unfair assumptions about the audiences background on some topics.
The thought XXX did a terrific job. All were very helpful, willing to slow their talks and present very well
XXX and XXX did a wonderful job of facilitation and engagement. Kudos!
In all the quality of instruction was excellent.
instruction was good but some cases I felt that instructors are presenting their research rather than topics and skills to be used in the class room. I believe both are useful but It is not clear how research presentations aligns with the course goal.

Q6 - To what extent would you agree with the following statements:

#	Question	Strongly Disagree	Disagree	Neither Agree nor Disagree	Agree	Strongly Agree	Total
1	Adequate assistance was available during workshop and small group sessions.	0	0	0	6	9	15
2	Overall, I was satisfied with the pre-course and course support provided by Jackson Lab staff.	0	0	0	2	13	15

Q7 - Please describe your goals for enrolling in this workshop.

Acquire skills and knowledge in genomics to apply in my courses.
I was hoping that we would work with big data, and learn strategies to deal with large datasets like parallelism, dimensional reduction, streaming or chunking data, and choosing the best AWS setups for a given dataset and analysis pipeline. Instead we used subsets that could be used in a classroom setting, which is useful for having students perform real-time analyses, but it makes it more 'cookbook' in nature. I was hoping I would be able to have students be able to perform novel analyses on full genomics/ '-omics' datasets to give my course a more discovery-based feel to it.
Learn new big data handling and analysis tools
Establish bioinformatics, genomics courses
To gain an entry point into a fast-moving field, both for teaching and research.
One of my main goals was for an update on current best practices and analysis pipelines for different types of data sets. I am training anywhere from 2-8 students a semester on RNAseq techniques and I want to make it as useful and relevant to them and this course helped significantly with that endeavor.
My goals were to learn enough to be able to teach an undergraduate-level bioinformatics course.
I hoped to gain an insight into the application of data science skills to genomics and how to better integrate relevant active learning modules within my courses. These goals were met.
hints and recommendations for teaching bioinformatics course mainly for computer science.
Gain more facility in programming and workflows. Develop more modules. Gain strategies for making work more accessible to undergrads.
I have few skills in these areas so it was very beneficial to get the opportunity for the education and the hands-on exercise. More, I was hoping for the chance to network with other educators.

Q8 - How can we improve this course and program?

Pre attendance homework for coding perhaps?
It might be better to have each module section co-taught by an experienced PUI genomics faculty member (pedagogical expert) paired with a JAX (content) expert in order to get the best of both worlds so to speak. The participants always looked at a tutorial in the context of being able to deliver that module to undergraduates pedagogically, rather than just understand and follow the workflow themselves. One or two of the tutorials were delivered in a way that frustrated many of the participants trying to follow along on their own laptops with the instructions, which indicated to me that that tutorial could not be taught to novice UG students in the same manner that the speaker presented it.
Having more defined modules. XXX's module was the highlight of the course in terms of organization and appropriate background information. Half baked modules are okay to learn a bit about a topic, but in most cases will not provide faculty (that are learning the content) enough confidence to actually implement it in the classroom.<br><br>It would also help to provide participants with some optional pre-workshop assignments associated with things like navigating R and command line navigation. Some participants like myself would of spent some time on this prior to the workshop, so that we could concentrate more on the content and use the time with the experts at the workshop more efficiently.
Scaffolding and deliberate sequencing of topics, with intro first then applications second.
Slow down on the hands on part.
Split in two.. beginning and intermediate with coding. It would be helpful to begin with software and package installation, even if we didn't use the particular instances that were installed in in-class activities.
more hands-on time, more group work to build networks and collaborations
The organization was truly excellent. However, there was a lot to cover within a week.
more focus on computer science part.
Help develop infrastructure for consortial work.

Q9 - Did you gain sufficient knowledge to launch a module or course at your institution focused on Genomics and Genomics Data Analysis? (Multiple answers are OK)

#	Answer	Count
1	Yes, Module	13
2	Yes, Course	6
3	Maybe, Module	0
4	Maybe, Course	6
5	No not yet	0
	Total	25

Q10 - What topics did we miss, what additional topics should we add?

(1.) Single cell genomics has exploded in the literature, so it would be interesting to learn about the complexities of experimental design, and data acquisition/analysis in this field.<br><br>(2.) XXX introduced it briefly in a lecture, but developing a *module* for machine learning in genomics (esp. using Python &/or Tensorflow) would be really exciting for the students.
Gene Finder Algorithms, HMMs
More small group deliberate discussion on how to incorporate topics.
some of the data visualization I think was cut out because of time or was not there to begin with. I think that would be very useful.
R was not sufficiently covered. Software and package installation was not sufficiently covered.
data analysis/data science and computational techniques.
Accessing SRA, interpreting networks, visualization, IT infrastructure
eQTL analysis would be very interesting.<br>Also, GWAS study with the hands on statistical analysis to interpret the data can be a nice short course.

Q11 - Are there topics we should remove from the course?

Although XXX is clearly a passionate world expert on database development, maintenance, and ontology, and although it is an important topic, I felt like the two hours could have been summed up in about 10 minutes with regard to the information that might be important for developing and teaching a undergraduate genomics/bioinformatics course.
Systems Biology
Combine the command line section with one of the other modules.
Thursday was the least relevant day for my needs and to me would have been better spent deepening the hands-on work for the other units.

Q12 - What do you think is the single largest barrier to undergraduate big data genomics education?

Training for professors.
fear about computers and math, logistics with IT are also a bit of a struggle
(1.) BIG DATA (storage, access, analysis, cost)! How can students analyze thousands human or other genomes, or single cell genomic datasets overnight or over a couple of days? Can they make novel big data genomics discoveries within a class?<br>(2.) Sometimes the biggest barrier is the University IT department!<br>(3.) Basic programming concepts and Math literacy can be problematic for some students.<br>(4.) Repeatedly pushing buttons, 'copy-&-pasting', or hitting 'Shift-Enter' can be boring for the students if the analysis is too "black box"/ abstracted away from what is actually happening under the hood.
Competency in use of the command-line and how quickly the field is moving.
Cross training if students to both biological relevance to be addressed via bioinformatic analysis
Faculty time and knowledge.
Feeling like I know enough to lead the students through the activities in a meaningful way. Also from experience at my institution- access to HPC and general internet connection issues makes things challenging.
faculty know-how
Unlike engineering students, most biology students do not receive training in computational methods for data analysis and the necessary math is not covered in the right context.
Administrative/IT support.
The need for the students to learn the language and underlying statistics and biology to adequately frame their questions. But, with some of the platforms we used (Galaxy and GeneWeaver, in particular) these become less of a barrier.

Q13 - I found the Canvas platform useful as a platform for sharing information and resources

#	Answer	Count
1	Strongly disagree	0
2	Somewhat disagree	0
3	Neither agree nor disagree	0
4	Somewhat agree	6
5	Strongly agree	9
	Total	15

Q14 - Will you continue to use the Canvas or SLACK webpages for accessing course and program resources for UG course development? (Multiple answers are OK)

#	Answer	Count
1	Yes, Canvas	3
2	Yes, SLACK	6
4	Yes, both	9
5	No	1
	Total	19

Q15 - Do you have any comments on how to more effectively mix hands-on work with didactic sessions?

Overall the balance was good. On Thursday, I believe we had 3 hours of back to back lectures, which was not optimal.
I thought it was a very good mix.
Perhaps scaffold more, and organize the lectures/hands on in a more deliberate way to ensure content builds on each session or at least has an intro first then application session.
Slow down during hands on work. Many times selections were being made or text was entered and I was trying to find things and missed what was happening. The XXX's module was excellent and I think that did a great job of going over basic command line material and you could move that earlier and then use the command line tutorial time for something else. Also I think it would be fine to give us homework and write in some of the code (especially in R) or a bash script before we came in the next day.
less coding and more prebuilt tools for beginners. There isn to enough time for both. More hands on activities and include time to work on the data that researchers have brought. Reduce the amount of time spent on talking about researchers' interests to increase time spent on bioinformatic tools and their application
every schedule block (monday morning, monday afternoon, etc) should have hands-on sessions--some schedule blocks this week (for instance thursday morning) were all lecture and felt less useful.
The biological applications/introductory material is very useful, but perhaps we could go through that in advance to save the time for hands-on workshop.
XXX and XXX did a fantastic job integrating the materials and hands on in different ways. The content was appropriately geared for what I was hoping to gain from the course (not too deep, based in the literature, and learning new skills). The hour-long lectures strung together without any hands-on to break it up were too dense and difficult to handle.
It was very well designed.

Q16 - Was the seminar content useful? - Usefulness of Content

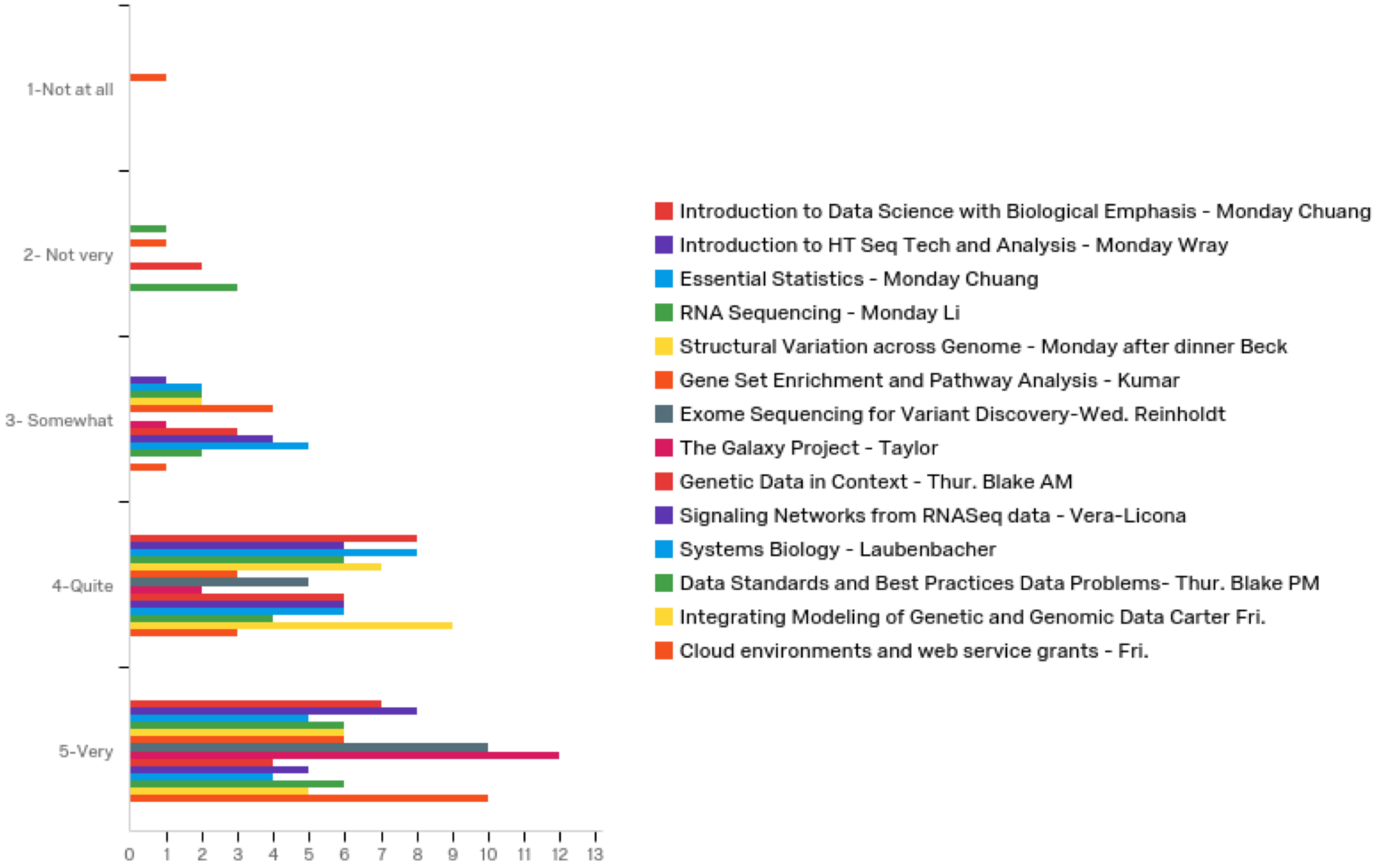

Q17 Was the Module useful and appropriate?

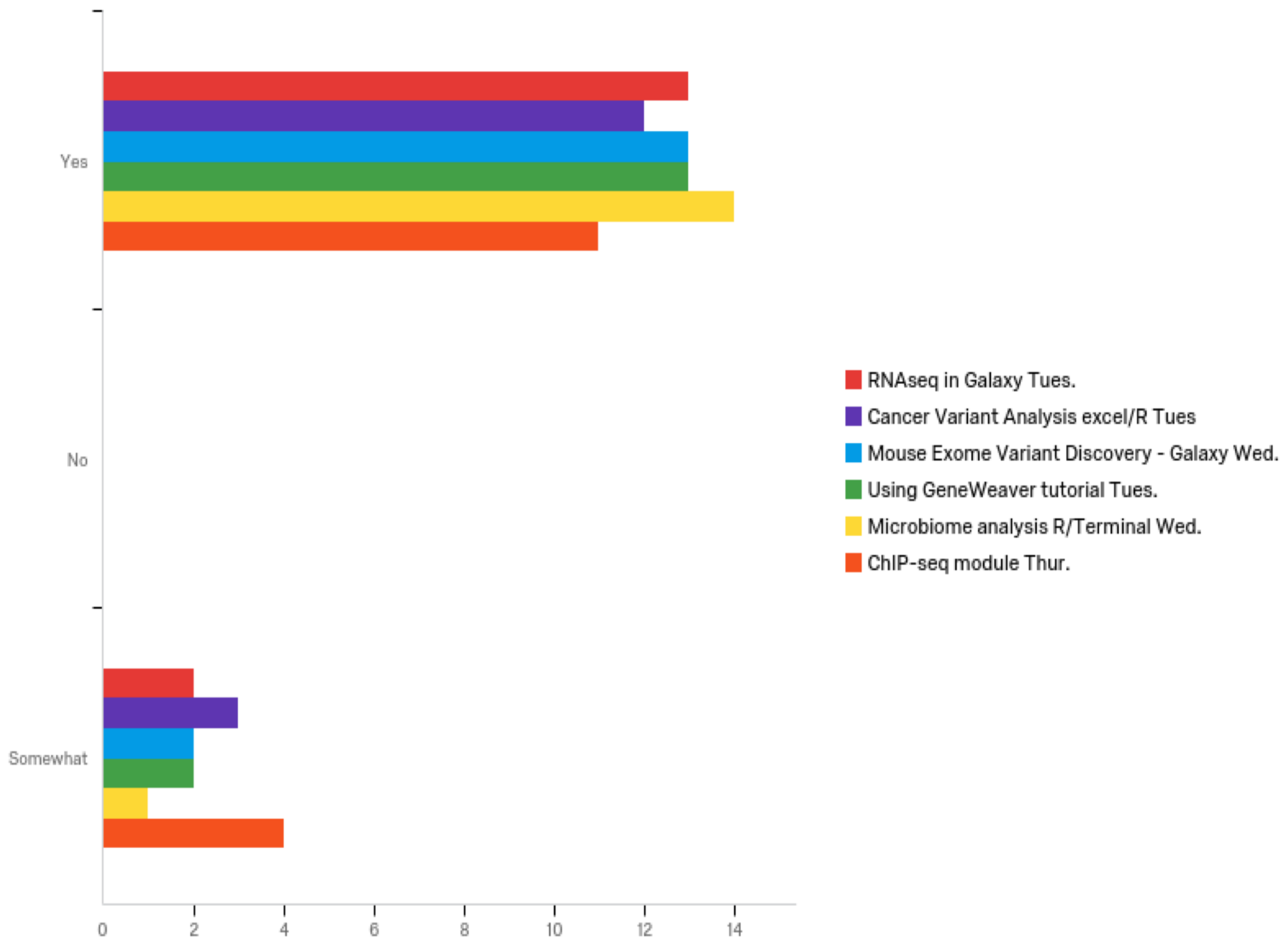

Q18 Will you use module in a course?

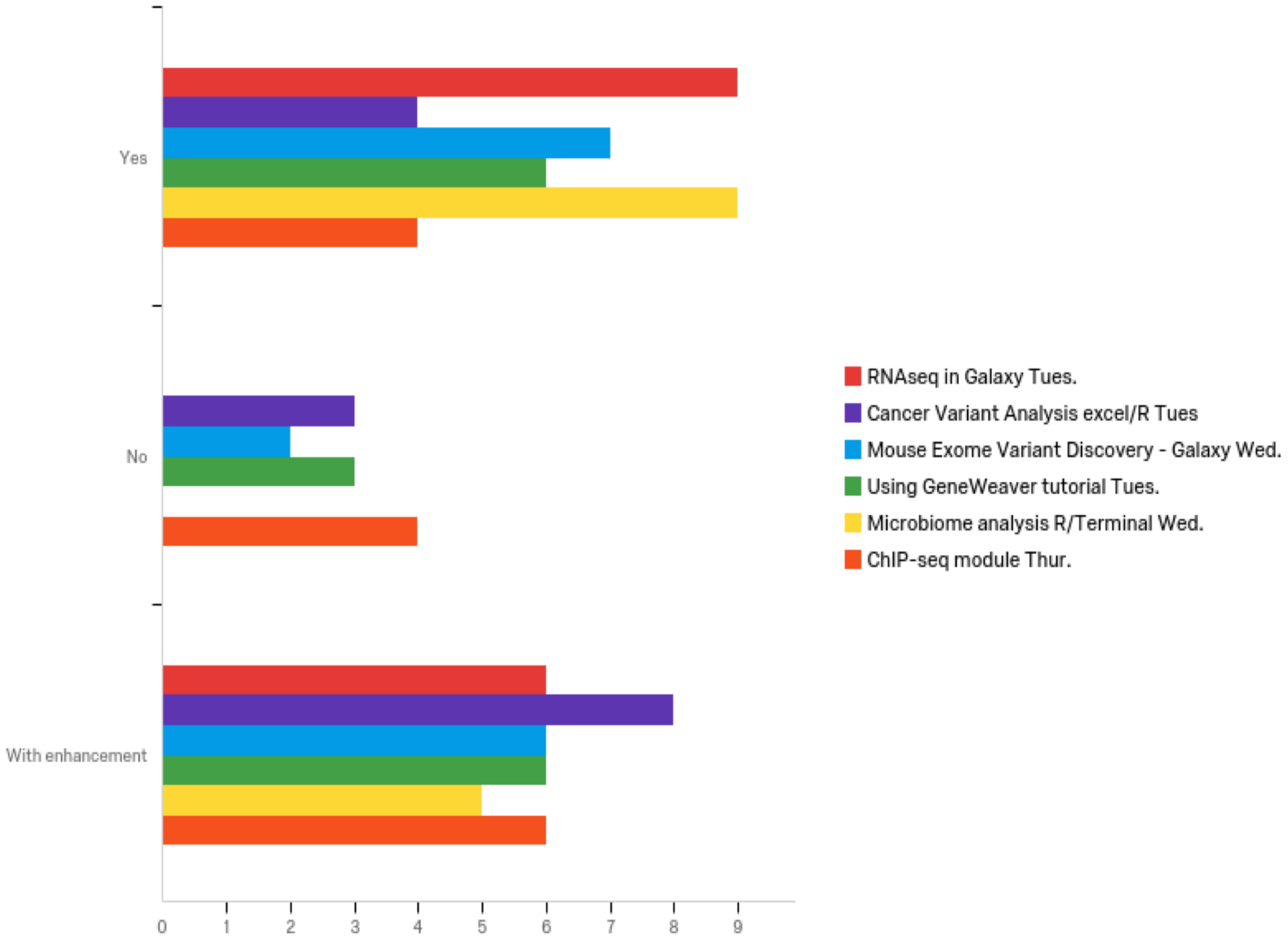

Q19 - Are there additional modules that JAX should develop and flesh out?

more time with existing units would have been great.<br>perhaps an evolution unit on building phylogenies?<br>perhaps a unit on how to get a new genome up and running?
I would like to see more of data analysis/science work. Also, more towards computer science work and how it is applied to biological problems.<br><br>Also, it would be useful to see how a research question be developed and what developing workflows.
microbiome<br>network analysis
eQTL analysis would be very interesting.<br>Also, GWAS study with the hands on statistical analysis to interpret the data can be a nice short course.

Q20 - How useful were interactive sessions? - Usefulness of Interactive Sessions

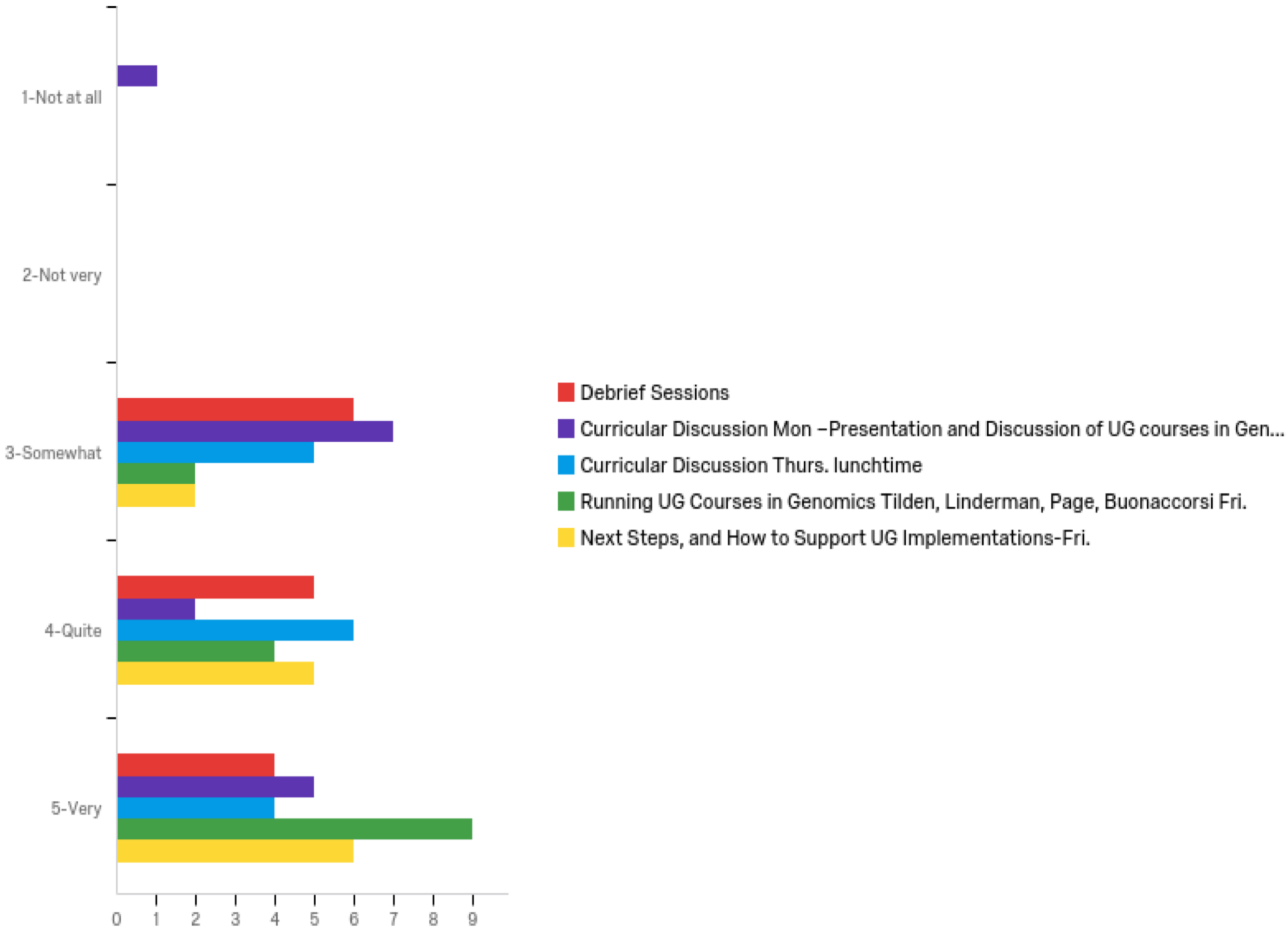

Q21 - Do you believe JAX created an inclusive environment for the program?

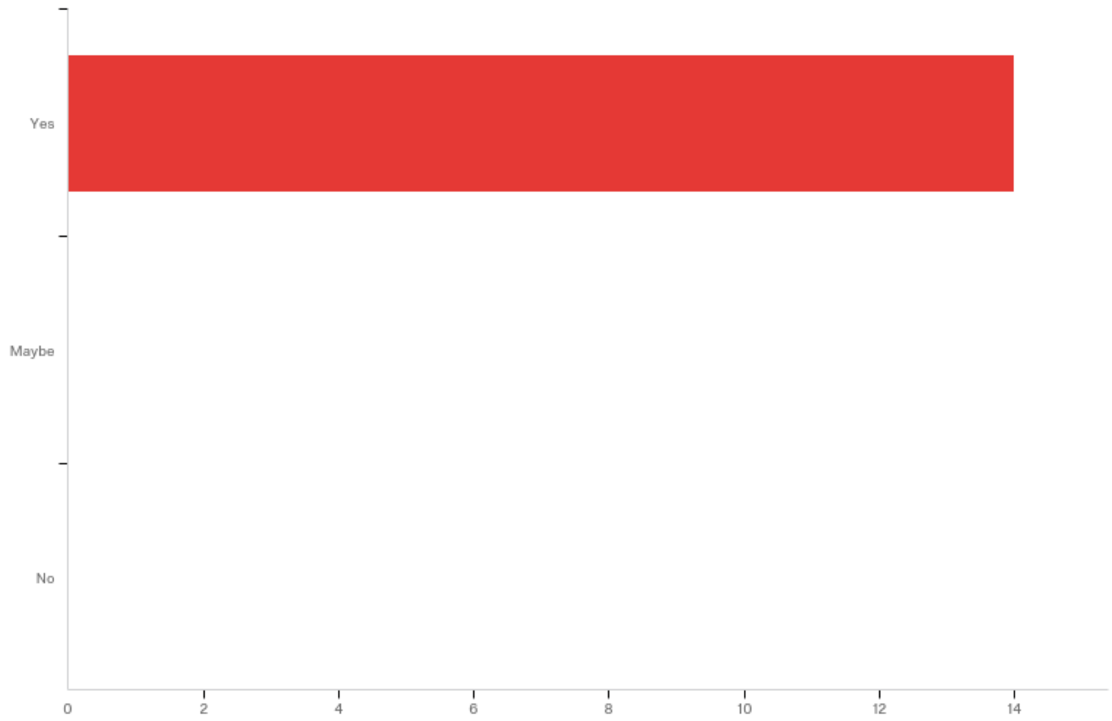

Q22 - Do you have any additional comments about the JAX environment, housing, hospitality or food?

Thank you for a great week! I learned lots of cool/relevant stuff.
All of this was excellent!
Excellent!
I thought it was a terrific experience. The hotel was convenient to Jax.
It was all lovely. Thank you.
The lunch choices were excellent. Thank you
JAX environment, housing, hospitality and food were all excellent.
hospitality and food was great. it would have been nice to organize happy hours in the evening at the hotel for those who might have wanted to hang out more. evenings were a little dead and there were not enough opportunities for socializing.
The hotel, meals, and communication regarding the course were all truly excellent.
Great stuff! THanks!
Amanda did a fantastic job with the logistics of food and lodging. I liked the variety of food over the different days and in particular the power bowls from the Monday lunch.<br><br>It would have been nice to have a group bus trip to someplace one of the evenings off because it felt very isolated without a vehicle.
