# Supplementary material for: Fostering bioinformatics education through skill development of professors: Big Genomic Data Skills Training for Professors
Source: PLoS Comput Biol. 2019 Jun 13;15(6):e1007026. doi: 10.1371/journal.pcbi.1007026 (PMC6563947; doi:10.1371/journal.pcbi.1007026)
Supplement: S4 File — This file contains all participating institutions to Big Genomic Data Skills Training for Professors from 2016 to 2018. (PDF) [file pcbi.1007026.s004.pdf]

JAX BD2K Participating Institutions*

Adams State University, CO
Agnes Scott College, GA
Albion College, MI
Baruch College, CUNY, NY
Bates College, ME
Bemidji State University, MN
Bethune-Cookman University, FL
Bucknell University, PA
Clark Atlanta University, GA
Colby College, ME
Dalhousie University, NS
DeSales University, PA
Eastern Kentucky University, KY
Eastern Virginia Medical School, VA
Franklin PIerce University, NH
Inter American University, PR
Jackson State University, MS
King University, TN
Lock Haven University, PA
Luther College, IA
Maine Medical Partners, ME
Meharry Medical College, TN
Metropolitan State University of Denver, CO
Middlebury College, VT
Minnesota State University Moorhead, MN
Moravian College, PA
Morehead State University, KY
Morgan State University, MD
New Mexico Highlands University, NM
New Mexico State University, NM
North Carolina Central University, NC
Northeastern State University, OK
Northern Kentucky University, KY
Northern Michigan University, MI
Northern New Mexico College, NM
Oakland University School of Medicine, MI
Oklahoma State University, OK
Prairie View A & M, TX
Salisbury University, MD
Salve Regina University, RI
Southern Arkansas University, AR
Southern Connecticut State University, CT
Southern Illinois Univ School of Medicine, IL
St. Edward's University, TX
SUNY Oswego, NY
Tennessee State University, TN
Trinity College, CT
Trinity Washington University, DC
U Maine Machias, ME
UCONN Health, CT
United States Air Force Academy, CO
University of Alaska Fairbanks
University of Arkansas for Medical Sciences
University of California, Irvine
University of Central Arkansas
University of Detroit Mercy, MI
University of Nevada, Reno
University of New England, ME
University of New Haven, CT
University of North Carolina at Pembroke
University of North Dakota
University of Puerto Rico
University of Puerto Rico at Cayey
University of Rhode Island
University of Saint Joseph, CT
University of South Florida
University of Tennessee at Chattanooga
University of Texas at Dallas
University of Texas-Rio Grande Valley
University of Wyoming
University of Vermont
Virginia Commonwealth University
Voorhees College, SC
Washington University, MO
Wayne State University School of Medicine, MI
Western Kentucky University, KY
Wheaton College, MA
Xavier University of Louisiana

Institutions in *italics* participating in a graduate level JAX BD2K workshop offering
